# Umbilical Cord Tissue-Derived Mesenchymal Stem Cells Induce T Lymphocyte Apoptosis and Cell Cycle Arrest by Expression of Indoleamine 2, 3-Dioxygenase

**DOI:** 10.1155/2016/7495135

**Published:** 2016-06-21

**Authors:** Xiuying Li, Zhuo Xu, Jinping Bai, Shuyuan Yang, Shuli Zhao, Yingjie Zhang, Xiaodong Chen, Yimin Wang

**Affiliations:** ^1^The Scientific Research Center, China-Japan Union Hospital, Jilin University, 126 Xiantai Street, Changchun, Jilin 130033, China; ^2^Rehabilitation Department, China-Japan Union Hospital, Jilin University, 126 Xiantai Street, Changchun, Jilin 130033, China; ^3^Shulanshi People's Hospital, Shulan, Jilin 132600, China; ^4^Eugenom Inc., 11107 Roselle Street, San Diego, CA 92121, USA; ^5^Research Service, Audie L Murphy Division, South Texas Veterans Health Care System, San Antonio, TX 78229-4404, USA; ^6^Department of Comprehensive Dentistry, University of Texas Health Science Center at San Antonio, San Antonio, TX 78229-3900, USA

## Abstract

It has been reported that human mesenchymal stem cells are able to inhibit T lymphocyte activation; however, the discrepancy among different sources of MSCs is not well documented. In this study, we have compared the MSCs from bone marrow (BM), adipose tissue (AT), placenta (PL), and umbilical cord (UC) to determine which one displayed the most efficient immunosuppressive effects on phytohemagglutinin-induced T cell proliferation. Among them we found that hUC-MSC has the strongest effects on inhibiting T cell proliferation and is chosen to do the further study. We observed that T lymphocyte spontaneously released abundant IFN-*γ*. And IFN-*γ* secreted by T lymphocyte could induce the expression of indoleamine 2, 3-dioxygenase (IDO) in hUC-MSCs. IDO was previously reported to induce T lymphocyte apoptosis and cell cycle arrest in S phase. When cocultured with hUC-MSCs, T lymphocyte expression of caspase 3 was significantly increased, while Bcl2 and CDK4 mRNA expression decreased dramatically. Addition of 1-methyl tryptophan (1-MT), an IDO inhibitor, restored T lymphocyte proliferation, reduced apoptosis, and induced resumption of the cell cycle. In addition, the changes in caspase 3, CDK4, and Bcl2 expression were reversed by 1-MT. These findings demonstrate that hUC-MSCs induce T lymphocyte apoptosis and cell cycle arrest by expressing abundant IDO and provide an explanation for some of the immunomodulatory effects of MSCs.

## 1. Introduction

Mesenchymal stem cells (MSCs) are a promising source of cells for cell-based therapeutics and regenerative medicine due to their ability to self-renew and differentiate into a number of functional cell types [1, 2]. To date, bone marrow-derived mesenchymal stem cells (BM-MSCs) have been the most widely studied family of stem cells. Despite their significant potential, the source(s) of these cells is limited and their number and quality decline with aging [3]. Recently, a number of laboratories have independently isolated multipotent stem cells from umbilical cord tissue that is capable of osteogenic, adipogenic, and chondrogenic differentiation [4]. Traditionally discarded after childbirth, the umbilical cord now appears to be an easily accessible and abundant source of stem cells. Further, the proliferative potential of human umbilical cord tissue-derived mesenchymal stem cells (hUC-MSCs) is greater than BM-MSCs [5]. Therefore, hUC-MSCs might be ideal for tissue regeneration in therapeutic applications.

Increasing evidence suggests that MSCs have the ability to suppress the activation of various immune cells, including dendritic cells and B- and T-lymphocytes [6–8].* In vitro*, MSCs are weakly immunogenic and suppress allogeneic T lymphocyte responses [9]. Also, they have been shown to alter the phenotype of natural killer cells and T lymphocytes, suggesting that transplantation of allogeneic MSCs may be clinically feasible [10]. It is possible that hUC-MSCs may generate an even smaller immune response than MSCs from adult tissues because of their fetal origin [11]. However, the mechanism(s) behind an immunosuppressive function for hUC-MSCs has not been fully elucidated [12, 13].

In order to find the best MSC sources from tissues, we screened the MSCs from bone marrow (BM), adipose tissue (AT), placenta (PL), and umbilical cord (UC) to determine which one displayed the most efficient immunosuppressive effects on phytohemagglutinin-induced T cell proliferation. Among them we found that UC-MSC have the strongest effects on inhibiting T cell proliferation and were chosen to do the further study.

A number of soluble factors produced by MSCs have been implicated in mediating their immunoregulatory activities, such as IFN-*γ* [14], transforming growth factor-*β*1 (TGF-*β*1), prostaglandin E_2_ (PGE_2_) [15], indoleamine 2,3-dioxygenase (IDO) [16], nitric oxide (NO) [17], and interleukin 10 (IL-10) [18]. In addition, it has been demonstrated that MSCs induce the arrest of cell division and apoptosis of activated T lymphocytes [19–22], but the mechanisms are not clearly defined. To understand the precise mechanisms behind the ability of hUC-MSCs to downregulate the immune response, we hypothesized that IDO, expressed by hUC-MSCs cocultured with T lymphocytes, induced cell-cycle arrest and apoptosis in T lymphocytes.

To test this hypothesis, we investigated the effect of hUC-MSCs on the activation of T lymphocytes, including proliferation, survival, and cytokine secretion. We attempted to define the cytokines involved by the use of inhibitors. We determined that hUC-MSCs suppress T lymphocyte proliferation by expressing IDO that stimulates T lymphocyte apoptosis and cell cycle arrest. This suppression by hUC-MSCs was abrogated by treating the cultures with 1-methyl tryptophan (1-MT), a potent competitive inhibitor of IDO.

## 2. Materials and Methods

### 2.1. Isolation and Expansion of MSCs

MSCs were isolated from 4 different sources: bone marrow (BM), adipose tissue (AT), placenta (PL), and umbilical cord (UC). The BM and AT samples were obtained from healthy volunteer donors, while the WJ and PL samples were obtained subsequent to a routine caesarean birth. All patients gave written informed consent and the study was approved by the Ethics Committee of the China-Japan Union Hospital at Jilin University. The age range of the donors was as follows: the BM, 18 to 43 years old, the AT, from 23 to 50 years, and the mothers, from 23 to 38 years old for umbilical cord and placenta. hUC-MSCs were isolated as previously described [23–25] with some modification. Briefly, collagenase and hyaluronidase (Sigma, USA) were used to digest the finely minced umbilical cord tissue after removal of the outer membranous layer of tissue. The digested cells were incubated in *α*-MEM medium supplemented with 10% fetal bovine serum (FBS; GIBCO, Australia) at 37°C in an atmosphere of 100% humidity and 5% CO_2_. After 48 h, nonadherent cells were removed by washing, and the medium changed twice a week until the cells reached approximately 80% confluence. BM-MSC, AT-MSC, and PL-MSC were isolated as previously described [26].

The cells were then detached by adding 0.05% trypsin and 0.02% EDTA (Sigma, USA) to the culture dish and incubating for 3 min at 37°C. The cells were then washed in fresh media, pelleted by centrifugation, and then resuspended and counted using a hemocytometer (Shanghai Qiujing, China). The resuspended cells were seeded into culture wells at 1000 cells/cm^2^.

### 2.2. Flow Cytometric Analysis of MSC

MSCs were harvested from culture flasks using trypsin-EDTA (as above) at 37°C and then washed twice with PBS. Next, cell suspensions were incubated with antibodies against CD44-phycoerythrin (PE), CD73-PE, CD14-PE, CD34-PE, CD90-fluorescein isothiocyanate (FITC), CD45-FITC, and CD105-PerCP, respectively (all from BD Biosciences, USA), and protected from light for 30 min at 4°C. After incubation, the cells were washed twice with PBS. Fluorescence intensity was measured using flow cytometry (Beckman Coulter FC500, USA) and the data were analyzed with CXP software (Beckman Coulter FC500).

### 2.3. MSCs Differentiation Potential

#### 2.3.1. Adipogenesis

MSCs were seeded into 12-well plates at 5 × 10^4^ cells/cm^2^ and grown in MSC growth medium (*α*-MEM with 10% FBS) at 37°C in a humidified atmosphere with 5% CO_2_ for a minimum of 2 h and up to 4 days (to near or complete confluence) before switching to adipogenesis-inducing medium (STEMPRO® Adipogenesis Differentiation Kit, GIBCO, USA). Cells were cultured in differentiation media for 21 days with changing media every 3 to 4 days. Adipocytes were observed by staining with oil red O (Sigma, USA) and visualized using a bright field microscope.

#### 2.3.2. Osteogenesis

MSCs were seeded into 12-well plates at 5 × 10^4^ cells/cm^2^ and grown in *α*-MEM containing 10% FBS at 37°C in a humidified atmosphere with 5% CO_2_. At confluence, the cells were treated with osteogenesis-inducing medium (STEMPRO® Osteogenesis Differentiation Kit, GIBCO, USA) for 35 days with changing media every 3 to 4 days. Mineral deposition, evidence of osteogenesis, was visualized using Alizarin Red S staining. Images were captured using a bright field microscope for qualitative analysis.

### 2.4. T Lymphocyte Proliferation Assay

Human T lymphocyte was purchased from JENNIO Biological Technology (Guangdong, China). T lymphocyte proliferation was induced by the addition of phytohemagglutinin (PHA; Sigma-Aldrich, USA) to the cultures. MSCs were inactivated by adding mitomycin C (10 *μ*g/mL) (MMC, Sigma–Aldrich, USA) to the cultures for 2 h at 37°C, followed by washing with PBS containing 10% FBS. Inactivated MSCs were cocultured with PHA-activated T lymphocytes at the indicated ratios in the 24-well tissue culture plates (contact culture) or transwells purchased from the MILLIPORE of USA (physically separated by a membrane) in growth media (*α*-MEM containing 10% FBS and 100 U/mL penicillin/streptomycin) for 5 days. 5-Bromo-20-deoxyuridine (BrdU) was then added to the cultures. After 18 hrs, proliferation was assessed using the BrdU-Assay Kit (Roche Applied Science, USA) according to the manufacturer's instructions.

### 2.5. Cell Apoptosis Assay

Apoptotic cells were detected using flow cytometry after staining with annexin-V-FITC/PI dual stain. After coculture, T lymphocytes were harvested, rinsed twice with PBS, and suspended in 500 *μ*L of binding buffer. The suspended cells were incubated for 15 min at 4°C with 5 *μ*L annexin V-FITC solution and then incubated for another 5 min at 4°C after adding 10 *μ*L of PI solution. The emitted green fluorescence of annexin-V (FL1) and red fluorescence of PI (FL2) were detected by a flow cytometer (Beckman Coulter Fc500, USA) with an excitation wave length of 488 nm and an emission wave length of 525 nm and 575 nm, respectively. For each sample, 10,000 events were recorded. The amount of early apoptosis, late apoptosis, and necrosis was determined as the percentage of annexin-V^+^/PI^−^, annexin-V^+^/PI^+^, and annexin-V^−^/PI^+^ cells, respectively.

### 2.6. Cell Cycle Assay

Cell cycle studies were performed using flow cytometric analysis as previously described [27]. Briefly, cocultured T lymphocytes were collected, washed, and suspended in cold 75% ethanol over night at 4°C. After fixation, cells were pelleted, washed with PBS, and stained using 50 *μ*g/mL propidium iodide (PI) and 50 *μ*g/mL RNase A (Beyotime, China) dissolved in 0.5 mL PBS. The suspension was protected from light and incubated for 30 min at 37°C. Then, the cells were analyzed by flow cytometry.

### 2.7. ELISA Assay

The supernatants from T lymphocyte-MSC cocultures (ratio is 1 : 1) and MSC cultures were collected at the time points of 24 h, 72 h, and 120 h. Each sample was in triplicate. Soluble IFN-*γ* in samples of the media was measured using a human IFN-*γ* ELISA Kit (Nanjing, Jiancheng, China), according to the instructions of the manufacturer.

### 2.8. IDO Inhibition Assay

hUC-MSCs and T lymphocytes were cultured using the Transwell system as previously described. The IDO inhibitor 1-methyl tryptophan (1-MT, Sigma, USA) was added to the cocultures at 250 *μ*m. The cells were cultured for 5 days and then proliferation, apoptosis, and cell cycle assays were performed.

### 2.9. Gene Expression Assay by Real-Time Quantitative RT-PCR in T Lymphocytes Cocultured with hUC-MSCs

Total cellular RNA was extracted using Trizol reagent (Life Technologies, USA) according to the manufacturer's instructions. RNA integrity was electrophoretically verified by ethidium bromide staining and OD260/OD280 nm absorption ratio (OD > 1.9). Total RNA (500 *μ*g) was reverse transcribed using 2.5 U AWV reverse trancriptase XL, 10 U RNase inhibitor, 1 mM dNTP mixture, 1.25 pmoL Oligo dT-Adaptor primer, 5 mM MgCl_2_, and 1 × RT buffer according to the manufacturer's instructions. A no amplification control (NAC) was included and did not contain reverse transcriptase. Reactions were incubated at 42°C for 30 min, then at 95°C for 5 min, and at 5°C for 5 min.

Real-time quantitative RT-PCR (qRT-PCR) was performed to assess gene expression for the following: Bcl2, caspase 3, and CDK4 in T lymphocytes cocultured with MSCs. Primers were designed using Primer3 Input (http://flypush.imgen.bcm.tmc.edu/primer/primer3_www.cgi) and synthesized with a *T*_*m*_ at 60°C. The primer sequences are listed in Table 1. QRT-PCR was conducted using an ABI 7500 FAST System (ABI Applied Biosystems, USA). 25 *μ*g of cDNA was used in qRT-PCR reactions with SYBR-Green PCR Master Mix (Applied Biosystems, Warrington, UK) and 0.2 *μ*m of gene-specific forward and reverse primers (Sangon Biotech, China). All PCR products demonstrated a single band by dissociation curve and gel electrophoresis. The thermocycler parameters for amplifying these genes were 1 cycle at 95°C for 10 min followed by 40 cycles at 95°C for 15 s, at 55°C for 15 s and at 72°C for 30 s. Beta-2-microglobulin (B2M) gene was used to normalize the cDNA amounts used in qRT-PCR. No template controls (NTC) and no amplification controls (NAC) were included in each reaction. Reactions were carried out in triplicate; the data were analyzed using the 2^−ΔΔCt^ method [28].

### 2.10. Western Blotting Analysis of IDO in hUC-MSCs Cocultured with T Lymphocytes

The protein samples were resolved by 10% SDS-PAGE and transferred onto polyvinylidene fluoride (PVDF) membranes (Millipore, Bedford, USA). After blocking and washing, the membranes were incubated with primary rabbit anti-human IDO antibody (Boster, China) and rabbit anti-human *β*-actin antibody (Abcam, USA), respectively. Following extensive washing, the membranes were incubated with goat peroxide- (HRP-) labeled anti-rabbit IgG (H + L) (Abcam, USA) and anti-mouse IgG (Abcam, USA) for 1 h in dark, respectively. After washing, an ECL detection system (ECL Kit; Beyotime, China) was used according to the manufacturer's instructions.

Quantification of the protein blotted was determined using Fuji film Intelligent Dark Box II and Image Gauge version 4.0 software.

### 2.11. HPLC-MS/MMS Detected the Activity of IDO

IDO enzyme activity of MSCs was measured by tryptophan-to-kynurenine conversion with photometric determination of kynurenine concentration in the supernatant as the readout [29]. In addition, IDO activity was quantified in T lymphocytes or cocultures. In coculture experiments, tryptophan and kynurenine concentrations were determined in cell culture supernatant by HPLC-MS/MMS. The supernatants from T lymphocyte-MSC cocultures (ratio is 1 : 1; adding or not adding 1-MT) and T lymphocyte cultures were collected. Then the tryptophan and kynurenine were detected by Beijing MS Medical Research Co., Ltd. The activity of IDO was showed by the percent of kynurenine according to the following formula: activity of IDO = Kyn/Try × 100%, where Kyn is the kynurenine concentration in MSC or cocultures and Try is the initial tryptophan concentration in the cultures.

### 2.12. Statistical Analysis

Statistical analyses were performed using SPSS software (SPSS, version 17.0, Chicago, IL, USA). Nonparametric test (Wilcoxon, Mann-Whitney) was used for statistical analysis. Parametric data are expressed as mean ± standard deviation (SD), with *n* = 3. Statistical significance was defined as *P* < 0.05.

## 3. Results

### 3.1. Characterization of the Cultured MSCs

The cell-surface antigen profiles of MSCs after 3 passages in culture were analyzed by flow cytometry. All MSCs tested positive for the expression of CD44, CD73, CD90, and CD105; they did not express CD14, CD34, and CD45 (data not shown), which is similar to the immunological phenotype of normal BM-MSCs.

When treated with adipocyte or osteoblast induction media, hMSCs differentiated into lipid vesicle-forming adipocytes that stained with oil red O or osteoblasts forming mineral deposits that stained with Alizarin, respectively (data not shown).

### 3.2. Comparison of T Lymphocyte Inhibition Potentials of 4 MSCs

**Figure 1:**
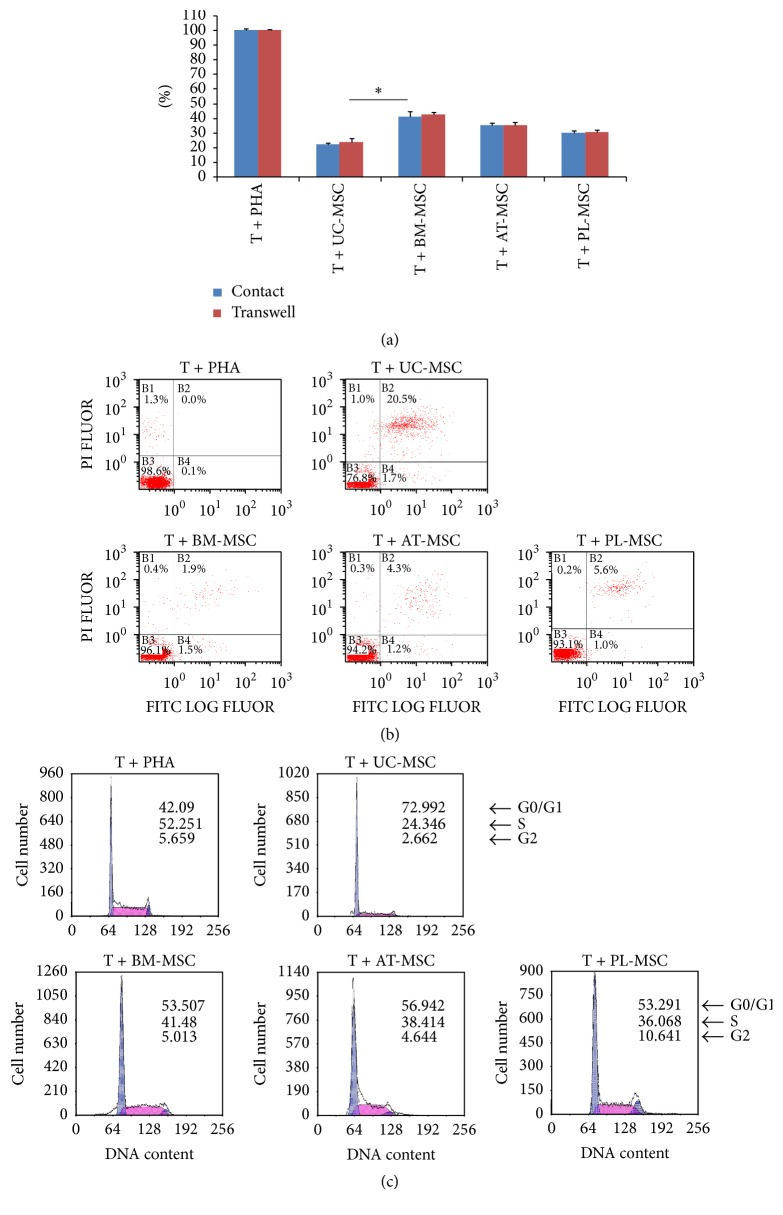
T lymphocyte inhibition potentials of 4 MSCs. (a) T lymphocytes proliferation was inhibited by MSCs in coculture. The data are expressed as the means ± SD from 5 independent experiments. The inhibitory effect of UC-MSC coculture groups on T lymphocytes proliferation was more prominent than that of the AT-, WJ-, and BM-MSC group. ^*∗*^ *P* < 0.05 when compared to the BM-MSC. (b) T lymphocyte apoptosis induced by MSCs in coculture. The apoptotic ratio of UC-MSC coculture groups on T lymphocytes was much highly prominent compared to that of the AT-, WJ-, and BM-MSC group. (c) G0/G1 phase arrest of T lymphocytes induced by MSCs in coculture. The arrest effect of the UC-MSC coculture groups on T lymphocytes was more prominent than that of the AT-, WJ-, and BM-MSC group.

T cell proliferation decreased significantly when cocultured with all four MSCs. The inhibitory effects of the UC-MSCs on T cell proliferation were the most prominent. *P* < 0.05 when compared to BM-MSC (Figure 1(a)). The apoptosis was observed on T cells when cocultured with 4 different MSCs. The apoptotic ratio was the highest on UC-MSC coculture group than that of the AT-, WJ-, and BM-MSC groups (Figure 1(b)). And also, G0/G1 phase arrest rate of T lymphocytes by UC-MSCs is highest in all four MSC coculture groups (Figure 1(c)).

### 3.3. UC-MSCs Inhibited the Release of IFN-*γ* of T Lymphocytes* In Vitro*

**Figure 2:**
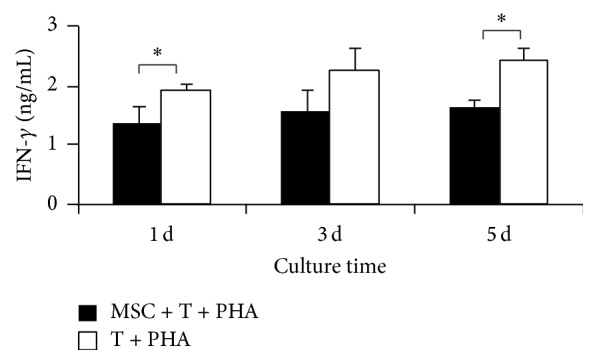
UC-MSCs inhibited the release of IFN-*γ* of T lymphocytes* in vitro*. T lymphocytes were stimulated with PHA (10 *μ*g/mL) during coculture with hUC-MSCs. The release of IFN-*γ* of T lymphocytes was measured by ELISA at 1 d, 3 d, and 5 d. Results are the average of 3 independent experiments. ^*∗*^ *P* < 0.05, compared to PHA-stimulated T lymphocytes controls.

The results of ELISA showed that T lymphocytes released a significant amount of IFN-*γ*, but the UC-MSCs did not release IFN-*γ*. And the UC-MSCs inhibited the release of IFN-*γ* of T lymphocytes* in vitro* (Figure 2).

### 3.4. IDO Was Associated with the Suppression of T Lymphocyte Proliferation

**Figure 3:**
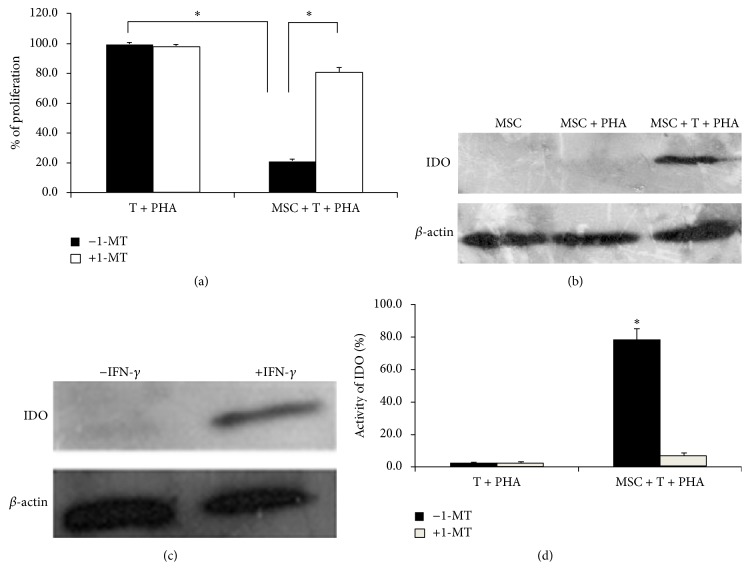
IDO, expressed by hUC-MSCs stimulated by IFN-*γ* in culture, was associated with the suppression of T lymphocyte proliferation. (a) T lymphocytes were stimulated with PHA (10 *μ*g/mL) during coculture with hUC-MSCs. T cell proliferation was measured by Brdu incorporation at 120 h. Results are the average of 3 independent experiments. ^*∗*^ *P* < 0.05, compared to coculture system that added 1-MT. (b) hUC-MSCs which were cocultured with T lymphocytes expressed protein level of IDO. (c) IFN-*γ* stimulated indeed the expression of IDO in UC-MSCs. (d) IDO activity was quantified in T lymphocytes or cocultures. Results are the average of 3 independent experiments. ^*∗*^ *P* < 0.05, compared to coculture system that added 1-MT.

To investigate the ability of hUC-MSCs to suppress T lymphocyte proliferation, cocultures were established using various ratios (1 : 1) of T lymphocytes to hUC-MSCs. As can be clearly seen in Figure 3(a), PHA activated T lymphocytes proliferation was significantly suppressed by UC-MSCs. IDO was expressed by hUC-MSCs which were cocultured with T cells. Western blot analysis demonstrates that human MSCs do not constitutively express IDO, but a substantial amount of IDO protein is induced by coculturing with T lymphocytes (Figure 3(b)). To confirm whether expression of IDO was stimulated by IFN-*γ*, 1.5 *μ*g/mL IFN-*γ* was added to the UC-MSCs cultures system. The results of western blotting displayed that IFN-*γ* stimulation is indeed needed for the expression of IDO in UC-MSCs (Figure 3(c)).  Figure 3(d) showed that the activity of IDO in the cocultures was higher than T lymphocytes cultures system.

To confirm whether IDO directly mediates the suppression of T lymphocytes proliferation, 250 *μ*m of 1-methyltryptophan (1-MT), an inhibitor of IDO, was added to the cocultures. In the treated cultures, T lymphocytes proliferation was almost completely recovered (Figure 3(a)).

### 3.5. hUC-MSCs-Induced T Lymphocyte Apoptosis Could Be Reversed by 1-MT

**Figure 4:**
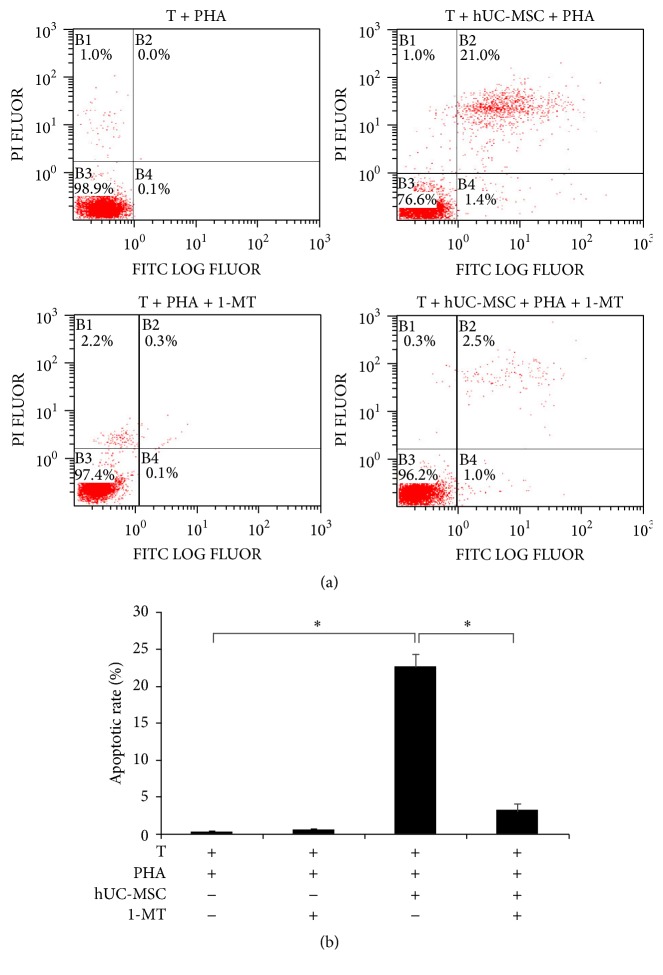
1-MT reverses the effect of hUC-MSCs on T lymphocyte apoptosis. Cultured T lymphocytes were stimulated for 120 h with PHA in the presence or absence of hUC-MSCs. 1-MT was included in some cultures. At harvest, the cells were stained with antibodies to annexin V and PI and analyzed by flow cytometry. (a) The FACS plots were representative of one of three experiments. (b) The results of three independent experiments were averaged. Bars represent the SD. ^*∗*^ *P* < 0.05.

To determine whether hUC-MSCs have the ability to induce apoptosis in activated T lymphocytes, cocultures of hUC-MSCs and T lymphocytes were examined using annexin V and PI double staining. As shown in Figure 4, there was a significant increase in the percentage of apoptotic cells in cultures where T lymphocytes were activated with PHA in the presence of hUC-MSCs. This result suggested that hUC-MSCs induced T lymphocyte apoptosis* in vitro*. The addition of 1-MT effectively rescued the cells from apoptosis, induced by hUC-MSCs, indicating that IDO played a role in T lymphocyte apoptosis.

### 3.6. hUC-MSCs-Induced T Lymphocyte Apoptosis through Downregulation of the Bcl-2/Caspase Pathway Which Could Be Reversed by 1-MT

**Figure 5:**
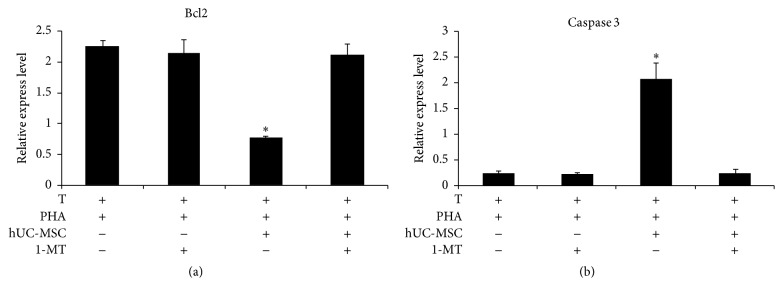
UC–MSCs suppressed Bcl-2 expression and induced caspase 3 expression during coculture with T lymphocytes. Cultured T lymphocytes were stimulated for 120 hours with PHA in the presence or absence of hUC-MSCs and treated with 1-MT. At harvest, the mRNA was isolated and subjected to RT-PCR for Bcl-2 (a) and caspase 3 (b). The data are presented as the means ± SD from 3 independent experiments. Bars represent the SD. ^*∗*^ *P* < 0.05.

The expression of Bcl2 and caspase 3 in T lymphocytes was detected by qRT–PCR to explore the mechanism of apoptosis caused by MSC. As expected, hUC-MSCs significantly inhibited mRNA expression of Bcl2 and promoted the expression of caspase 3 (Figure 5). The addition of 1-MT effectively reversed the changes in Bcl2 and caspase 3 expression induced by the hUC-MSCs.

### 3.7. Cell Cycle Arrest by IDO, through Downregulation of CDK4

**Figure 6:**
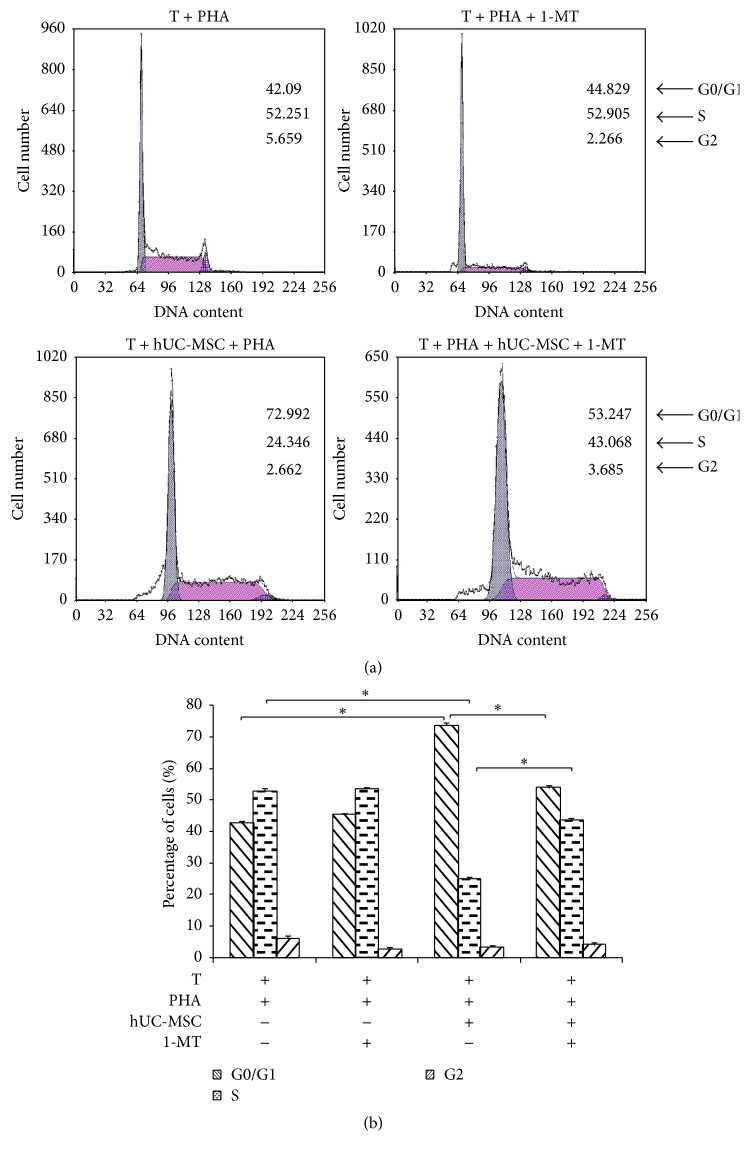
1-MT reverses T lymphocytes cell cycle arrest induced by hUC-MSCs. Cultured T lymphocytes were stimulated for 120 h with PHA in the presence or absence of hUC-MSCs and 1-MT. At harvest, the DNA content of the cells was measured by PI staining using flow cytometry. (a) The FACS plots were representative of one of three experiments. (b) The results of three independent experiments were averaged. Bars represent the SD. ^*∗*^ *P* < 0.05.

When hUC-MSCs were cocultured with T lymphocytes, cell cycle analysis revealed that G0/G1 phase was increased to 72.992 ± 1.002% from 42.090 ± 0.928% and S phase was decreased to 24.346 ± 0.984% from 52.251 ± 1.053%. The percentage of T lymphocytes in G0/G1 phase was decreased to 53.247 ± 1.003% by addition of 1-MT, while the percentage of T lymphocytes in S phase increased to 43.068 ± 0.812%, which was significantly different from the control group (52.251 ± 1.053%; Figure 6). These data indicate that T lymphocytes were arrested in G0/G1 phase when cocultured with hUC-MSCs. This arrest was partly reversed by 1-MT, indicating that IDO secreted by hUC-MSCs played a major role in producing this effect.

**Figure 7:**
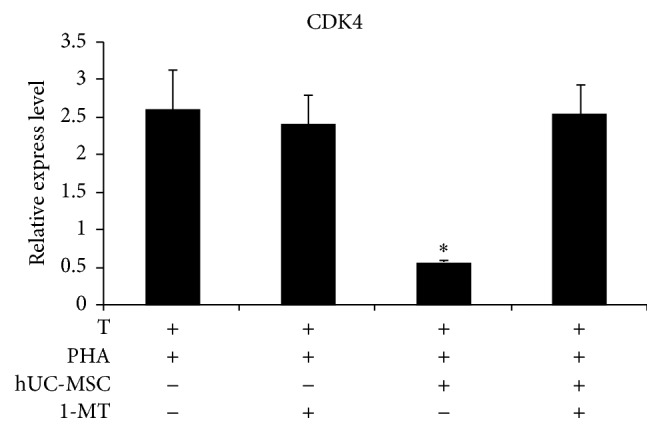
UC–MSCs suppressed CDK4 expression in cocultured T lymphocytes. Cultured T lymphocytes were stimulated for 120 h with PHA in the presence or absence of hUC-MSCs and 1-MT. At harvest, the mRNA was isolated and subjected to RT-PCR to assess changes in CDK4 expression. The data are presented as the means ± SD from 3 independent experiments. Bars represent the SD. ^*∗*^ *P* < 0.05.

Based on the above findings, we further analyzed changes in mRNA expression of cyclin-dependent kinase 4 (CDK4), a member of the cyclin-dependent kinase family. As shown in Figure 7, the presence of hUC-MSCs significantly inhibited CDK4 expression. After addition of 1-MT, the expression of CDK4 by T lymphocytes was almost completely restored.

## 4. Discussion

MSCs were originally isolated and characterized from bone marrow. However, BM-MSCs are not readily available as their quantity and capacity for proliferation and differentiation decrease with age [3]. In contrast, human umbilical cord tissue is easily obtained and often discarded as biological waste after parturition. MSCs derived from umbilical cord exhibit an increased capacity for proliferation and differentiation, as well as lower immunogenicity, as compared to BM-MSCs [5, 30].

In order to find a best MSC source for clinical use, we screened 4 MSCs from bone marrow, adipose tissue, placenta, and umbilical cord and found that UC-MSC has the strongest potential to suppress T lymphocyte proliferation. Thus, hUC-MSCs have emerged as a promising new resource for cell-based therapeutic applications. Although it has been reported that hUC-MSCs suppress T lymphocyte activation [20], the mechanism of this effect remains unclear. In current study, we have obtained data suggesting that hUC-MSCs potently suppress T lymphocyte proliferation by directing them into cell-cycle arrest (G0/G1 phase) and apoptosis. These results are consistent with observations reported by others [20]. More importantly, we also showed that hUC-MSCs expressed enormous amounts of the enzyme IDO. It is known that IDO degrades tryptophan in the microenvironment resulting in T lymphocyte apoptosis [31]. In our study, we added 1-MT, a potent competitive inhibitor of IDO, to coculture and demonstrated that it blocked the suppressive effects of hUC-MSCs on T lymphocyte activation by restoring proliferation (i.e., switching G0/G1 phase to S phase) and attenuating T lymphocyte apoptosis.

It has been reported that BM-MSCs prevent T lymphocyte activation by preventing the cells from entering into S phase [32]. Here, we also examined whether the cell cycle of activated T lymphocytes was affected by hUC-MSCs. In the presence of hUC-MSCs, the number of T lymphocytes in S phase was greatly reduced in response to soluble factor production by hUC-MSCs. Cyclin dependent kinases (CDKs) are a family of protein kinases which regulate different stages of the eukaryotic cell cycle [33–35]. Since CDK4 plays a central role in the regulation of the G0/G1 phase and is required for the G1/S phase transition, we measured mRNA expression of CDK4 in a coculture system and found that hUC-MSCs downregulated CDK4. The addition of 1-MT to the cocultures restored CDK4 expression in T lymphocytes and significantly increased the number of T lymphocytes in S phase. These results further demonstrated that hUC-MSCs suppressed the proliferation of activated T lymphocytes by driving them into G0/G1 phase.

In the present study, we further demonstrated that induction of T lymphocyte apoptosis involves members of the Bcl-2 family, including antiapoptosis gene products (e.g., Bcl-2) and proapoptosis gene products (e.g., Bax) [36, 37]. Bax proteins induce apoptosis by promoting the release of mitochondrial cytochrome c. Countering the effect of Bax, Bcl-2 inhibits the release of mitochondrial cytochrome c and apoptosis. In the current study, we show that hUC-MSCs induce T lymphocyte apoptosis by downregulating Bcl-2 and upregulating caspase 3. The addition of 1-MT to the cocultures reversed the downregulation of Bcl-2 and caspase 3 and significantly reduced T lymphocyte apoptosis.

Recent studies support the idea that, in addition to defense against pathogens, IDO participates in the regulation of T lymphocyte responses and suppression T lymphocyte proliferation [38]. It has been speculated that expression of IDO in antigen presenting cells of the immune system may control autoreactive T lymphocytes [38]. Consequently, IDO plays an important role in the development of autoimmune disease, organ transplant rejection reaction, and cancer. Recent reports suggested that IDO-facilitated conversion of tryptophan to kynurenine not only depleted tryptophan from the cellular environment but also resulted in production of kynurenine downstream metabolites that mediated inhibition of T cell proliferation [10]. In this study, hUC-MSCs were able to inhibit T lymphocyte proliferation owing to the large amount of IDO expressed by stem cells. When the IDO inhibitor 1-MT was added to the cultures, the inhibitory effect of IDO on T lymphocytes was mostly reversed.

It is possible that factors may be secreted by hUC-MSCs, such as IL-10, IFN-*γ*, TGF-*β*1, PGE_2_, and NO, and have an effect on T lymphocyte activation. The fact that the addition of 1-MT to cocultures of hUC-MSCs and T lymphocytes only restored proliferation to 80% of the uninhibited level suggests that other factors have effects too.

In conclusion, our findings provide insight into the mechanism(s) employed by hUC-MSCs to strongly suppress T lymphocyte activation. By expressing abundant amounts of IDO induced by IFN-*γ*, the stem cells target the T lymphocyte cell cycle and apoptosis pathways. Based on these results and the fact that the source of hUC-MSCs is virtually unlimited, this study supports the possibility of using hUC-MSCs as a source of trophic factors for treating graft-versus-host disease (GVHD) as well as other autoimmune diseases.

## Figures and Tables

**Table 1:** Primer sequences and product sizes.

Symbol	Primer sequences	Product sizes (bp)
Bcl2	F-CGGTGGGGTCATGTGTGTG R-CGGTTCAGGTACTCAGTCATCC	90
Caspase 3	F-GAGTGCTCGCAGCTCATACCT R-CCTCACGGCCTGGGATTT	81
CDK4	F-GGAGGAGGAGGTGGAGGA R-GTCCATCAGCCGGACAAC	105
B2M	F-CTATCCAGCGTACTCCAAAG R-GAAAGACCAGTCCTTGCTGA	188
