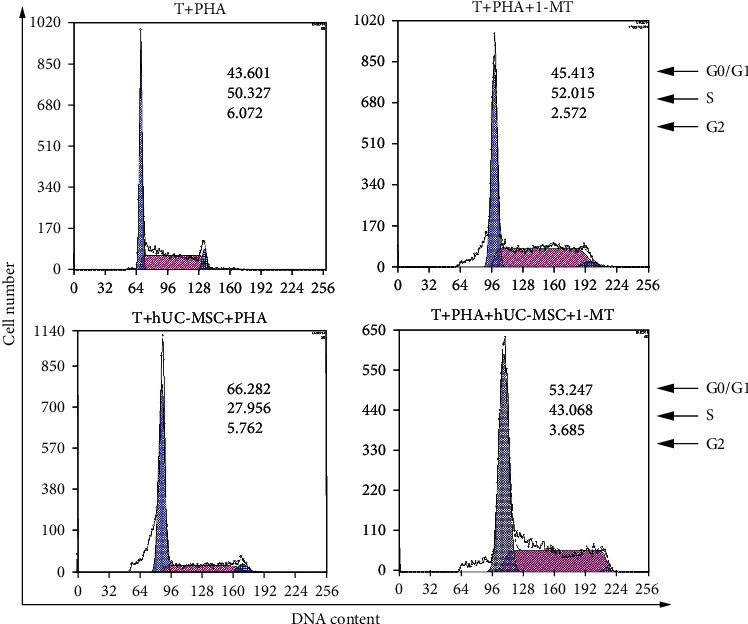# Corrigendum to “Umbilical Cord Tissue-Derived Mesenchymal Stem Cells Induce T Lymphocyte Apoptosis and Cell Cycle Arrest by Expression of Indoleamine 2, 3-Dioxygenase”

**DOI:** 10.1155/2021/9817948

**Published:** 2021-04-16

**Authors:** Xiuying Li, Zhuo Xu, Jinping Bai, Shuyuan Yang, Shuli Zhao, Yingjie Zhang, Xiaodong Chen, Yimin Wang

**Affiliations:** ^1^The Scientific Research Center, China-Japan Union Hospital, Jilin University, 126 Xiantai Street, Changchun, Jilin 130033, China; ^2^Rehabilitation Department, China-Japan Union Hospital, Jilin University, 126 Xiantai Street, Changchun, Jilin 130033, China; ^3^Shulanshi People's Hospital, Shulan, Jilin 132600, China; ^4^Eugenom Inc., 11107 Roselle Street, San Diego, CA 92121, USA; ^5^Research Service, Audie L Murphy Division, South Texas Veterans Health Care System, San Antonio, TX 78229-4404, USA; ^6^Department of Comprehensive Dentistry, University of Texas Health Science Center at San Antonio, San Antonio, TX 78229-3900, USA

In the article titled “Umbilical Cord Tissue-Derived Mesenchymal Stem Cells Induce T Lymphocyte Apoptosis and Cell Cycle Arrest by Expression of Indoleamine 2, 3-Dioxygenase” [1], the authors identified that the incorrect image was presented in Figure 6(a). The images of T + PHA group in Figure 6(a) and Figure 1(c) were repeated. The authors apologize for this error and explained that the error occurred during manuscript preparation and confirm that it does not affect the results and the conclusions of the article. The corrected Figure 6(a) is as follows:

## Figures and Tables

**Figure 1:**